# Relationship among Short and Long Term of Hypoinsulinemia-Hyperglycemia, Dermatophytosis, and Immunobiology of Mononuclear Phagocytes

**DOI:** 10.1155/2015/342345

**Published:** 2015-10-11

**Authors:** Thais F. C. Fraga-Silva, Camila M. Marchetti, Luiza A. N. Mimura, Gisele A. Locachevic, Márjorie A. Golim, James Venturini, Maria S. P. Arruda

**Affiliations:** ^1^Department of Biological Sciences, School of Sciences, Universidade Estadual Paulista (UNESP), 17033-360 Bauru, SP, Brazil; ^2^Department of Microbiology and Immunology, Institute of Biosciences of Botucatu, Universidade Estadual Paulista (UNESP), 18618-970 Botucatu, SP, Brazil; ^3^Department of Clinical, Toxicological and Bromatological Analysis, School of Pharmaceutical Sciences of Ribeirão Preto, University of São Paulo (USP), 14040-903 Ribeirão Preto, SP, Brazil; ^4^Botucatu Blood Center, Universidade Estadual Paulista (UNESP), 18618-970 Botucatu, SP, Brazil

## Abstract

Dermatophytes are fungi responsible for causing superficial infections. In patients with diabetes mellitus (DM), dermatophytosis is usually more severe and recurrent. In the present study, we aimed to investigate the influence of short and long term hypoinsulinemia-hyperglycemia (HH) during experimental infection by *Trichophyton mentagrophytes* as well as alterations in the mononuclear phagocytes. Our results showed two distinct profiles of fungal outcome and immune response. Short term HH induced a discrete impaired proinflammatory response by peritoneal adherent cells (PAC) and a delayed fungal clearance. Moreover, long term HH mice showed low and persistent fungal load and a marked reduction in the production of TNF-*α* by PAC. Furthermore, while the inoculation of TM in non-HH mice triggered high influx of Gr1^+^ monocytes into the peripheral blood, long term HH mice showed low percentage of these cells. Thus, our results demonstrate that the time of exposure of HH interferes with the TM infection outcome as well as the immunobiology of mononuclear phagocytes, including fresh monocyte recruitment from bone marrow and PAC activity.

## 1. Introduction

Dermatophytes are fungi responsible for superficial infections in skin, hair, and nails [1]. Dermatophytosis affects approximately 20–25% of the world's population [2–4] and* Trichophyton rubrum* and* Trichophyton mentagrophytes* (TM) are the most common causative agents [4, 5]. Patients with diabetes mellitus (DM) are particularly susceptible to this fungal infection [6–8]. While fingernail due to TM is observed in 30.8% of type I DM, nondiabetic patients exhibit prevalence of 4.54% [9].

Several physiologic and metabolic disorders, such as peripheral vascular disease, peripheral neuropathy, poor glycemic control, obesity, and hypertriglyceridemia, are associated with high rates of infection in DM patients [10]. Simultaneously, DM triggers several changes in the immune response, including an unbalanced macrophage activity [11]. Although monocytes/macrophages from onset type I DM as well as from experimental diabetic murine models showed a proinflammatory profile, characterized by intense production of hydrogen peroxide (H_2_O_2_) and release of TNF-*α* [12, 13], this profile is not able to prevent mycotic infections in DM patients. The interaction of* T. rubrum* conidia with nonactivated resident peritoneal macrophages leads to downregulation of costimulatory molecules expression, such as CD80 and CD54, and phagocytosis but induces production of TNF-*α* and IL-10 [14]. Thus, the balance between intense proinflammatory response observed in DM and the modulation of macrophage activity triggered by dermatophytes remains to be clarified.

Recently, at least two subsets of human and murine monocytes have been recognized according to their functions under inflammatory and steady-state conditions [15]; these subsets can be identified by the expression of the surface molecules CD14 and CD16 in humans (classical CD14^+^CD16^−^ and nonclassical CD14^+^CD16^+^ monocytes) [16] and Ly6C/Ly6G (Gr1) in mice (resident CD115^+^Gr1^−^ and inflammatory CD115^+^Gr1^+^ monocytes) [17, 18]. The murine CD115^+^Gr1^+^ monocyte subset expresses high levels of the adhesion molecule E-selectin (CD62L) and CC-chemokine receptor 2 (CCR2) [18]. According to Landsman et al. [19], this monocyte subset migrates between the blood and the bone marrow and is only found in other locations when recruited as a result of inflammation. CD115^+^Gr1^−^ monocytes differ by their absent/low Gr1 receptor expression and higher fractalkine receptor (CX3CR1) expression [17]; this monocyte subset exhibits blood vessel patrolling behavior and may serve as precursor to alternatively activated macrophages during tissue repair and resident macrophage/dendritic cells (DCs) turnover [20]. A recent report has shown that type I DM patients exhibit an expansion of the pool of nonclassical CD14^+^CD16^+^ monocytes [21]; however, the consequence of this expansion and their potential use as a biomarker of inflammation must be further elucidated.

In a previous study we observed that hyperglycemic-hypoinsulinemic (HH) mice infected with* T. mentagrophytes* showed a delay in the fungal clearance after 14 days after infection [22]. This result was associated with decreased counts of peripheral blood CD4^+^T cells [22]. In a present study, we hypothesized that during the early infection progress, when HH and normal mice exhibit similar fungal burden, alterations in the innate immune response could be associated with the delay in the outcome of dermatophyte-infected HH mice. Thus, we aimed to evaluate the peritoneal adherent cells activity as well as the distribution of peripheral blood monocyte subsets in HH mice infected with* T. mentagrophytes*. Furthermore, we investigated the effect of time of HH exposition in these mice.

## 2. Methods

### 2.1. Mice

Two-month-old male, weight-matched, Swiss mice purchased from CEMIB/UNICAMP (Campinas, São Paulo, Brazil) were maintained at the Animal House of the* Laboratório de Imunopatologia Experimental, LIPE* (UNESP, Bauru, SP, Brazil). The mice were housed in groups of three to five and were provided with food and water* ad libitum*. The experimental protocol was performed in accordance with the ethical principles for animal research adopted by the National Council for the Control of Animal Experimentation (CONCEA). This study was approved by the Ethical Committee of School of Sciences (UNESP, Bauru, SP, Brazil).

### 2.2. Fungi

The* Trichophyton mentagrophytes* (TM) strain (2118/99-ILSL) was isolated from a human lesion and obtained from the fungal collection of the Lauro de Souza Lima Institute, Bauru, São Paulo State, Brazil, and was maintained by frequent subculture on a Mycosel agar (Difco Laboratories, USA) slant at 25°C in our laboratory.

### 2.3. Induction of HH Condition Using Alloxan Administration

Alloxan administration to laboratory animals selectively destroys insulin-producing pancreatic *β*-cells [22, 23] without promoting pancreatitis [24, 25]. Mice were intravenously administered alloxan (Sigma Chemical Co., St. Louis, MO) in a single dose of 60 mg kg^−1^ of body weight injected into the caudal vein. Hyperglycemia was confirmed 48 h later using Accu-Chek Advantage II blood glucose test strips (Roche, Mannheim, Germany). Only mice showing blood glucose levels >200 mg dL^−1^ were considered as having HH and included in the experiment. The glucose levels during the experiment were typically 400–600 mg dL^−1^ in HH mice. In the present study, the success rate of alloxan-induced HH was approximately 80–90%. Control mice (TM and CTL) presented serum glucose levels ranging from 90 to 180 mg dL^−1^.

### 2.4. Fungal Inoculum

The strain was cultured on Mycosel agar slants for 10 days at 25°C. The fungi were washed carefully with a sterile saline solution. The fungal suspension was then mixed twice for 10 s on a vortex mixer and decanted for 5 min. The supernatants were collected and washed twice [26]. Fungal viability was determined by cotton blue staining, and the concentrations were adjusted to 5 × 10^8^ viable TM conidia per mL. The mice were subcutaneously injected in the footpad, with 0.04 mL of TM inoculum (HHTM and TM groups). Noninfected groups (HH and CTL groups) were treated identically with a sterile saline solution.

### 2.5. Experimental Designs

**Figure 1:**
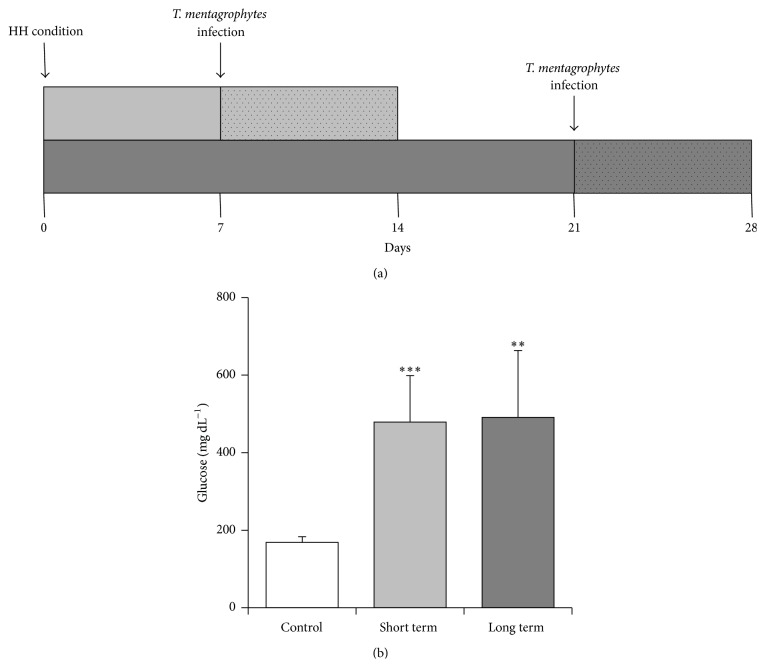
Experimental design. Mice were intravenously inoculated with alloxan (60 mg kg^−1^) into the caudal vein. Hyperglycemia was confirmed after 48 h by blood glucose levels >200 mg dL^−1^. (a) Mice that were exposed to HH condition for 7 days constituted the HH group (short term) and those for 21 days constituted the HH group (long term). Mice from HH groups (short and long term) were infected with 2 × 10^7^ *T. mentagrophytes* microconidia into footpad. (b) Glucose levels (mg dL^−1^) after 7 days (short term) and 21 days (long term) of HH installation. The results are expressed as mean ± SD (*n* = 6–12/group). Unpaired *t*-test, *p* < 0.05.  ^*∗*^ *p* < 0.05 and  ^*∗∗*^ *p* < 0.01 indicate statistical differences among HH group* versus* the control group (uninfected/HH-free mice).

The study was performed using two distinct experimental designs according to the time of HH exposure: 7 (short term) and 21 (long term) days after HH induction (Figure 1). Each experiment included four subgroups as follows: (1) HHTM group, composed of HH mice inoculated with TM; (2) HH group, composed of HH mice and noninfected mice; (3) TM group, composed of non-HH mice and inoculated with TM; (4) control group, composed of non-HH mice and uninfected mice. All mice were submitted to the same procedures and sterile saline solution was used as placebo control. The mice were euthanized at 1, 2, and 7 days after fungal challenge, that is, on 8, 9, and 14 (short term) days and 22, 23, and 28 (long term) days after HH installation. The study was performed at least in two independent experiments and 5 or 6 mice were enrolled in each subgroup per time point.

### 2.6. Collection of the Biological Material

Mice were euthanized by CO_2_ inhalation, and fresh peripheral blood was collected by cervical decapitation into a tube containing ethylenediaminetetraacetic acid (EDTA) as an anticoagulant. Tissue was then dissected from the footpad, popliteal lymph nodes, liver, spleen, and kidneys and submitted to microbiological analyses.

### 2.7. Colony-Forming Unit (CFU) Determination

Fragments of the collected organs were weighed and homogenized in 1 mL of PBS, and 0.1 mL of the homogenate was cultured on 15 × 90 mm Mycosel agar plates at 25°C for 14 days. Each sample was assessed in duplicate. Total colonies were counted, and the results were expressed as the CFU number (log_10_) of TM per gram of tissue.

### 2.8. Peritoneal Adherent Cell Culture

After euthanasia, mouse abdominal skin was removed, and peritoneal cell suspensions were obtained by washing the peritoneal cavity with 10 mL of sterile ice-cold phosphate-buffered saline (PBS) in pH 7,4. The suspension was then centrifuged and the cells were resuspended in 1.0 mL of RPMI-1640 (Nutricell, Campinas, SP, Brazil) supplemented with 10% heat-inactivated fetal calf serum (Gibco BRL, Grand Island, NY, USA), penicillin (100 IU mL^−1^), and streptomycin (100 mg mL^−1^) (Gibco). The cell concentration was adjusted to 2.0 × 10^6^ mononuclear phagocytes mL^−1^ as judged by the uptake of 0.02% neutral red. The peritoneal cells were placed in 96-well flat-bottomed microtiter plates (Costar, Cambridge, MA, USA) and incubated for 2 hours at 37°C and 5% CO_2_ in a humidified chamber to allow peritoneal cells to adhere and spread. Nonadherent cells were removed by washing the wells 3 times with RPMI-1640, and the remaining adherent cells (>95% mononuclear phagocytes as assessed by morphological examination) were used for experiments. The peritoneal adherent cells (PAC) were cultured at 37°C and 5% CO_2_ in supplemented RPMI-1640. After 24 hours, the cell-free supernatants were harvested and stored at −80°C pending cytokine analysis.

### 2.9. Measurement of Hydrogen Peroxide Release (H_2_O_2_)

PAC (2 × 10^6^ cells mL^−1^) obtained as described before were maintained in RPMI-1640 culture medium at 37°C and 5% CO_2_ for 24 h. At the end of the cell culture period, the supernatant was removed and macrophages were incubated with phenol red solution (dextrose (Sigma), phenol red (Sigma), and horseradish peroxidase type II (Sigma)) and plated at 37°C in 5% CO_2_ for 1 h according to the methods of Russo et al. [27]. The reaction was stopped with the addition of 1 N NaOH and the H_2_O_2_ concentration was determined using a chemiluminescence microreader (ELx 800; BioTek Instruments Inc., Winooski, VE, USA).

### 2.10. Quantification of Nitric Oxide (NO) Production 

To determine NO levels, the production of nitrite (a stable end product of NO) was measured in the cell-free supernatants of PAC cultured according to the methods of Green et al. [28]. Briefly, 0.1 mL of cell-free supernatant was incubated with an equal volume of Griess reagent containing 1% sulfanilamide (Synth, Diadema, SP, Brazil), 0.1% naphthalene diamine dihydrochloride (Sigma), and 2.5% H_3_PO_4_, at room temperature for 10 min, and the nitrite accumulation was quantified using an chemiluminescence microreader (ELx 800; BioTek Instruments Inc., Winooski, VE, USA). The concentration of nitrite was determined using sodium nitrite (Sigma) diluted in RPMI-1640 medium as a standard.

### 2.11. Cytokine Analyses

The levels of TNF-*α* and IL-10 were measured in the cell-free supernatants using a cytokine Duo-Set Kit (R&D Systems, Minneapolis, MI, USA), according to the manufacturer's instructions. Each sample was analyzed in duplicate.

### 2.12. Determination of Peripheral Blood Monocyte Subsets

Blood monocyte subsets, obtained as described above, were analyzed for differences in the profile expression of surface molecules by flow cytometry using a fluorescence-activated cell sorter (FACS) according to Breslin et al. [29]. The following monoclonal antibodies were purchased from BioLegend (San Diego, CA, USA): Alexa Fluor 488-conjugated rat IgG2b anti-mouse Ly-6G/Ly-6C (Gr1), clone RB6-8C5; phycoerythrin- (PE-) conjugated rat IgG2a anti-mouse CD115 (CSF-1R), clone AFS98; allophycocyanin- (APC-) conjugated rat IgG2b anti-mouse CD45, clone 30-F11; and rat IgG2a and rat IgG2b isotype controls. Flow cytometry was performed using a FACSCalibur (BD), and the data were analyzed using FlowJo software (Tree Star).

### 2.13. Statistical Analysis

To test for the normality of data, results were analyzed by Shapiro-Wilk's test. Comparisons between two samples were made by unpaired *t*-test and more than three samples were made by one-way ANOVA followed by Tukey's test for parametric variables and by Kruskal-Wallis followed by Dunn's test for nonparametric variables. The data were analyzed using the software GraphPad Prism 5 (San Diego, CA, USA) and values of *p* < 0.05 were considered statistically significant.

## 3. Results

### 3.1. Long Term HH Mice Infected with TM Exhibit Persistent Viable Fungi

**Figure 2:**
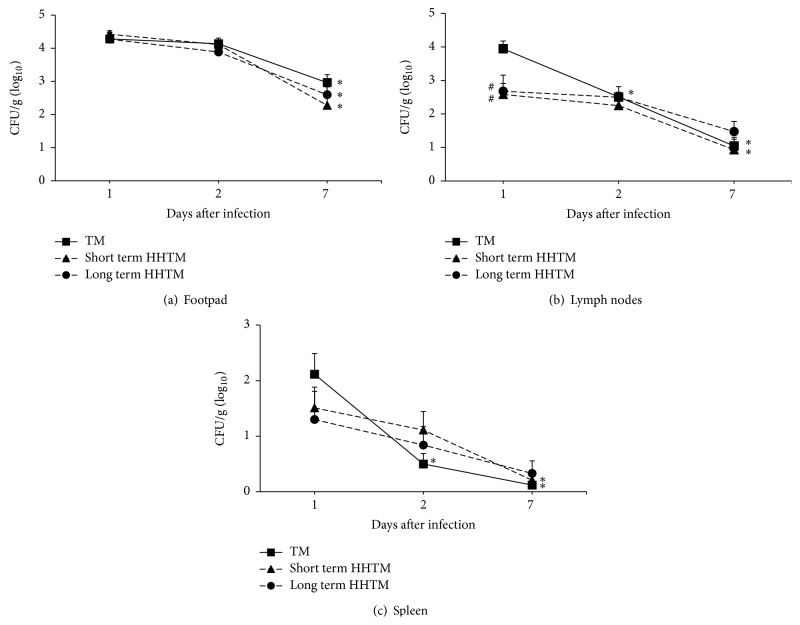
Microbiological evaluation. Swiss mice were infected with* T. mentagrophytes* and fungal load was evaluated 1, 2, and 7 days after infection in the footpads (a), popliteal lymph nodes (b), and spleen (c). The results are expressed as mean ± SD (*n* = 6–12/group) of the CFU (log_10_⁡) per gram of tissue. Unpaired *t*-test, *p* < 0.05.  ^*∗*^Statistical difference to day 1 and  ^#^statistical difference between TM and HHTM groups in the same time point.

As observed in Figure 2, the viable fungi were recovered from all evaluated organs, including the footpad, popliteal lymph nodes, and spleen. After 7 days after infection (p.i.) all groups exhibited lower fungal load compared to day 1 in footpad samples (Figure 2(a)). The popliteal lymph nodes (Figure 2(b)) and spleen (Figure 2(c)) fungal burden decreased after 2 days p.i. in non-HH TM infected mice (TM group) and decreased after 7 days p.i. in TM and short term HHTM groups, compared with fungal burden at day 1 p.i. The fungal load was lower in popliteal lymph nodes samples of short and long term HH infected groups compared to TM group (Figure 2(b)) at day 1 p.i.

### 3.2. *T. mentagrophytes* Clearance Is Associated with Early Release of Fresh Blood Inflammatory Monocytes and Regulated Macrophage Activity

**Figure 3:**
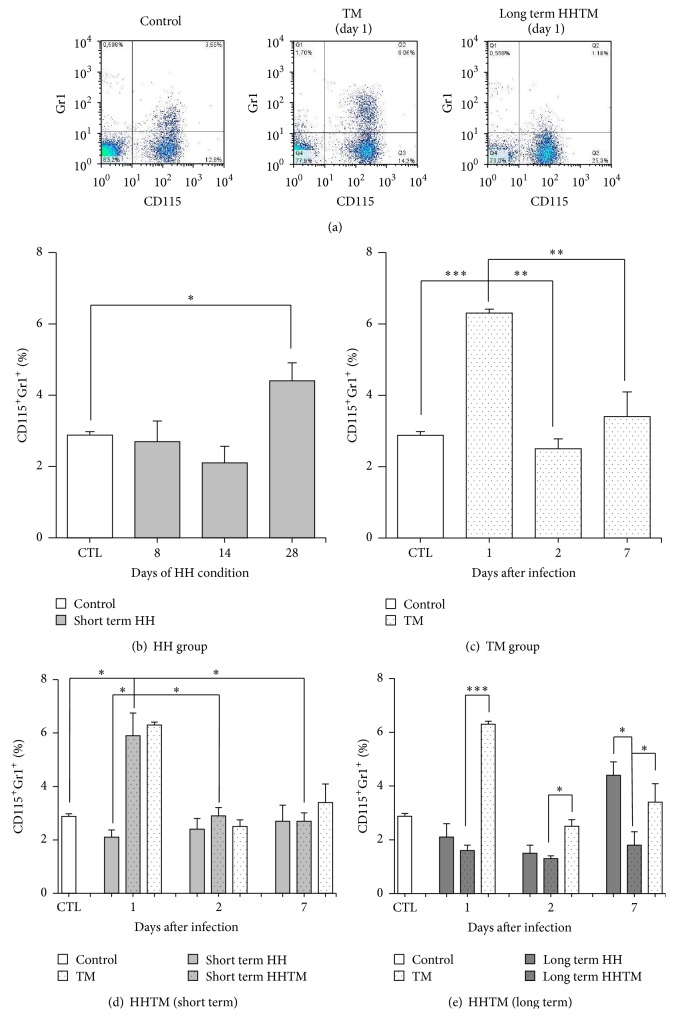
The distribution of peripheral blood monocyte subsets in TM-infected mice and noninfected HH mice. (a) Representative flow cytometry dot plot of data acquisition showing CD115 and Gr1 surface expression on peripheral blood cells. The numbers in the dot plots represent the percentages of each cell population within the square. (b) The distribution of inflammatory CD115^+^Gr1^+^ monocytes in the HH group. Peripheral blood cells were analyzed from 8 to 28 days after HH induction. (c) The distribution of inflammatory CD115^+^Gr1^+^ monocytes in the TM group. Peripheral blood cells were analyzed at 1, 2, and 7 days after fungal challenge. (d) The distribution of inflammatory CD115^+^Gr1^+^ monocytes in the short term HHTM group. (e) The distribution of inflammatory CD115^+^Gr1^+^ monocytes in the long term HHTM group. Data are expressed as the mean ± SEM of percentages and are representative of two independent experiments (*n* = 6 mice/group). Multiple comparisons of means were performed using ANOVA with the Tukey* post hoc* test (^*∗*^ *p* ≤ 0.05).

**Figure 4:**
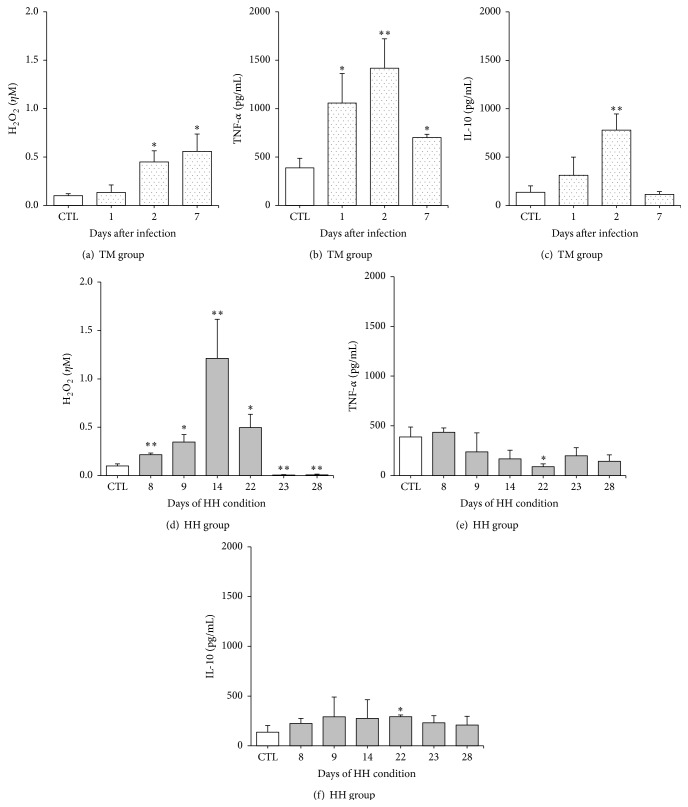
Kinects of hydrogen peroxide (H_2_O_2_), tumor necrosis factor- (TNF-) *α*, and interleukin- (IL-) 10 production by PAC from mice infected with* T. mentagrophytes* and HH mice. The results are expressed as mean ± SEM (*n* = 5-6/group). Unpaired *t*-test, *p* < 0.05.  ^*∗*^ *p* < 0.05 and  ^*∗∗*^ *p* < 0.01 indicate statistical differences between each experimental time point* versus* the control group (uninfected/HH-free mice).

As expected, 1 day after fungal inoculation, we observed in the TM group an increased percentage of CD115^+^Gr1^+^ monocytes in the peripheral blood (Figures 3(a) and 3(c)). At 2 days p.i., when the fungal load started to decrease, the levels returned to normal. At this time point (2 days p.i.) the peritoneal adherent cells of the TM group showed high levels of TNF-*α*, IL-10, and H_2_O_2_ (Figures 4(a)–4(c)). On day 7, the levels of TNF-*α* and H_2_O_2_ remained higher than the control group (Figures 4(a)–4(c)).

### 3.3. Long Term of HH Exposure Increases the Numbers of Total Blood Monocytes and Gr1^+^ Monocytes and Decreases the Production of H_2_O_2_ and TNF-*α* by Peritoneal Adherent Cells

Next, we evaluated the influence of HH condition* per se*, that is, in noninfected mice. Although there was no difference in glucose levels between short and long term HH mice (Figure 1), we observed increased percentage of Gr1^+^ monocytes on day 28 (Figure 3(b)). The HH-induced mice showed high production of H_2_O_2_, reaching a peak at the 14th day; afterwards, the levels started to decrease, and from the 23rd day the production was lower than the control group (Figure 4(d)). The levels of TNF-*α* remained unaltered in the short term of HH condition; from day 22 (long term), the levels were lower than the control group (Figure 4(e)). The IL-10 levels are the same in all experimental times points in the short term of HH condition; from day 22 (long term), the levels were higher than the control group (Figure 4(f)).

### 3.4. *T. mentagrophytes* Challenge in HH Long Term Reduces Percentage of Peripheral Blood Gr1^+^ Monocytes and Decreases the Production of TNF-*α* by Peritoneal Adherent Cells

**Figure 5:**
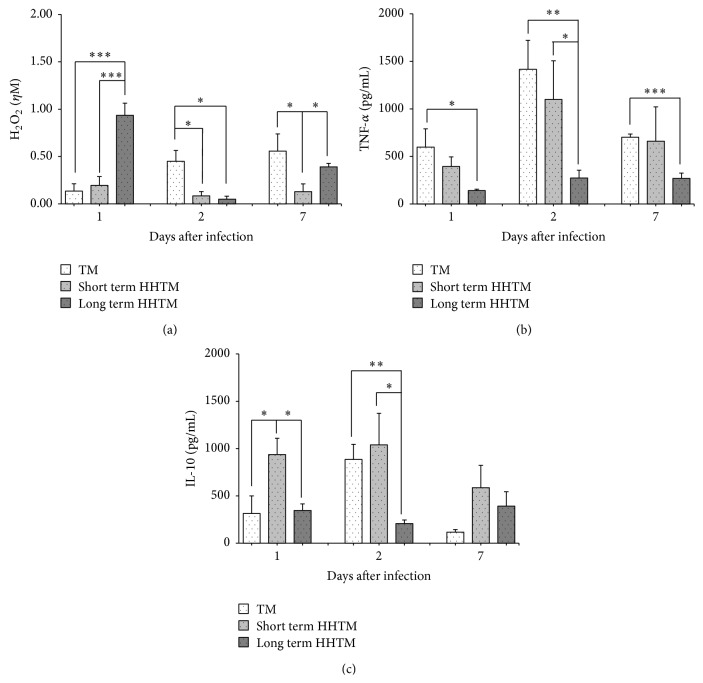
The hydrogen peroxide (H_2_O_2_) (a), tumor necrosis factor- (TNF-) *α* (b), and interleukin- (IL-) 10 (c) production. Peritoneal adherent cells were analyzed at 1, 2, and 7 days after fungal challenge. Data are expressed as the mean ± SEM and are representative of two independent experiments (*n* = 5-6 mice/group). The comparison between TM and HHTM groups was performed using unpaired *t*-test (^*∗*^ *p* ≤ 0.05,  ^*∗∗*^ *p* ≤ 0.01, and  ^*∗∗∗*^ *p* ≤ 0.001).

As for distribution of peripheral blood monocyte subsets, we observed that mice of HHTM long term group showed lower percentage of Gr1^+^ monocytes on day 1 p.i. than TM group (Figure 3(e)). We also observed an increased production of H_2_O_2_ at 1 day p.i. by PAC only in the long term HHTM group compared to the TM and long term HHTM groups (Figure 5(a)). However, at 2 days p.i. both short term and long term HHTM groups showed a lower H_2_O_2_ production in comparison to TM group. At 7 days long term HHTM group showed a lower H_2_O_2_ production in comparison to TM and long term HHTM groups (Figure 5(a)). The long term HHTM group showed a TNF-*α* decrease in comparison to TM group in all periods analyzed (Figure 5(b)). Additionally, at 2 days p.i. the long term HHTM group showed a TNF-*α* decrease in comparison to short term HHTM group (Figure 5(b)). Our results demonstrate an IL-10 increase at 1 day p.i. in short term HHTM group compared to the TM and long term HHTM groups (Figure 5(c)). The long term HHTM group showed an IL-10 decrease at 1 and 2 days p.i. compared to short term HHTM group and showed an IL-10 decrease at 2 days p.i. compared to TM group (Figure 5(c)). No differences were observed at 7 days p.i. among the groups.

## 4. Discussion

In a previous report, we have demonstrated that HH alloxan-induced mice exhibit a delay in the* T. mentagrophytes* clearance [22] that mimics one important aspect of the severity of dermatophytosis in DM patients. However, chronic dermatophytosis, subclinical infection, and recurrence episodes, also observed in DM patients, as well as immune-related mechanisms involved in these processes remain to be elucidated. Here, we extend our findings and aim to evaluate the initials immune-related mechanisms involved in* T. mentagrophytes* (TM) infection associated with different time of HH exposure. For this, we focused on evaluating the peritoneal adherent cells (PAC) activity and the distribution of peripheral blood monocyte subsets in HH mice inoculated with TM during short and long term state of HH condition.

Interestedly, soon after fungal inoculation (day 1), we observed that the fungal load in the popliteal lymph nodes was lower in the HHTM groups. According to Moriguchi et al. [30], the high blood levels of glucose present in HH mice lead to increased local lymphatic drainage and impaired dextran particles retention in the lymph nodes. Thus, the loss of the particle retention ability of the lymph nodes could explain the lower fungal load in HHTM mice. Moreover, the TM group showed downfungal burden at 2 days p.i. in internal organs while in short term HHTM the fungal burden decreased at 7 days and in the long term HHTM the fungal burden remains. Our findings demonstrated that, in the long term HH mice, TM infection is more discrete but persistent. These results indicate a possible defect in the innate immunity of DM patients and reinforce the hypothesis that internal organs could act as a reservoir for dermatophytes [31].

With regard to monocytes subsets, a large number of newly induced Gr1^+^ cells are released from the bone marrow into the blood stream and these cells enter the infected tissue and participate in the initial phase of the inflammatory response in short time, thereafter differentiating into proinflammatory macrophages and TNF-producing DCs [32, 33]. The high percentage of this monocyte observed on day 1 after fungal challenge underlines that also* T. mentagrophytes* are able to trigger this response. Even in the presence of fungi on day 2, the percentage of peripheral blood Gr1^+^ monocytes decreased to the baseline percentage. Uninfected HH mice exhibited high percentages of peripheral blood total monocytes and Gr1^+^ monocytes only at 28 days after HH induction. A recent study demonstrated that streptozotocin- (STZ-) induced diabetes mice develop several changes in the bone marrow after 12 months, including increased numbers of inflammatory CCR2^+^ monocytes that can “home” to the target tissues of chronic diabetic complications, such as the retina and the kidney [34]. Although our results are in agreement with those of Hazra et al. [34], the experimental model of alloxan-induced HH goes beyond because it permits the evaluation of two HH-related alterations: short term HH (HH* per se*) and chronic disturbances evoked by long term HH. Thus, the results observed at 28 days after HH induction in mice with alloxan-induced HH appear to be more related to the severe damage caused by chronic HH disturbances.

Our results showed that fungal infections processes in the early phase of HH (*per se*) did not alter the distribution of peripheral blood monocyte subsets. On the other hand, the long term HHTM mice showed lower percentage of Gr1^+^ monocytes rather than the expected increase. This phenomenon could be related to at least three mechanisms: (1) impaired CCR2-mediated recruitment from the bone marrow; (2) increased migration from the blood stream to the bone marrow or to the sites of infection/inflammation; (3) fast differentiation to Gr1^−^ monocytes.

It has been described that hyperglycemia induces intracellular ROS directly, mainly by AGE/RAGE interactions [35–37]. Our results exhibited the same profile; HH mice showed an increased production of H_2_O_2_ until day 22 after HH condition; however, in the HHTM groups the production of H_2_O_2_ decreased on the second day p.i. Considering that H_2_O_2_ is a potential fungicidal against* T. mentagrophytes *[38] and* T. rubrum *[39], the lower production of H_2_O_2_ probably contributed to fungal delay and/or persistence. Furthermore, we observed that long term HH leads to a reduced production of TNF-*α* even during fungal challenge. Similarly, peritoneal macrophages with “M2-like” phenotype have also been recently identified in animals with prolonged DM [11, 40]. Besides a proinflammatory response, a concomitant and regulated immune response is a crucial step to eliminate pathogens. Antigens of* T. rubrum* trigger IL-10 and TNF-*α* production by peritoneal macrophages [14]. Also* in vivo* experiments showed simultaneous release of these cytokines that was associated with protective response [26]. In the present study, mice from long term HHTM group showed low levels of IL-10 and TNF-*α*, suggesting that short and long term exposition to HH condition result in different degrees of unbalanced PAC activity.

Briefly, our study showed that (1) short and long term HH interfered with the dynamic of fungal dissemination to the internal organs; (2) long term HH affected the response of mononuclear cells by increasing the percentages of total monocytes and Gr1^+^ monocytes and the production of H_2_O_2_ but decreasing TNF-*α* production by peritoneal adherent cells; (3) long term HHTM mice showed a lower percentage of peripheral blood Gr1^+^ monocytes and decreased TNF-*α* production by peritoneal adherent cells. Our findings go beyond the fungal-host relationship in a HH milieu and bring new immunological descriptions in dermatophytosis and in the alloxan-induced HH mice.

## References

[1] Hainer B. L. (2003). Dermatophyte infections. *The American Family Physician*.

[2] Murray P. R., Rosenthal K. S., Kobayashi G., Pfaller M. A. (1997). *Microbiology Medical*.

[3] Havlickova B., Czaika V. A., Friedrich M. (2008). Epidemiological trends in skin mycoses worldwide. *Mycoses*.

[4] Kaur R., Kashyap B., Bhalla P. (2008). Onychomycosis—epidemiology, diagnosis and management. *Indian Journal of Medical Microbiology*.

[5] Gürcan Ş., Tikveşli M., Eskiocak M., Kiliç H., Otkun M. (2008). Investigation of the agents and risk factors of dermatophytosis: a hospital-based study. *Mikrobiyoloji Bulteni*.

[6] Muller L. M. A. J., Gorter K. J., Hak E. (2005). Increased risk of common infections in patients with type 1 and type 2 diabetes mellitus. *Clinical Infectious Diseases*.

[7] Gupta S., Koirala J., Khardori R., Khardori N. (2007). Infections in diabetes mellitus and hyperglycemia. *Infectious Disease Clinics of North America*.

[8] Parada H., Veríssimo C., Brandão J. (2013). Dermatomycosis in lower limbs of diabetic patients followed by podiatry consultation. *Revista Iberoamericana de Micología*.

[9] Macura A. B., Gasińska T., Pawlik B., Obłoza A. (2007). Nail susceptibility to fungal infection in patients with type 1 and 2 diabetes under long term poor glycaemia control. *Przegla̧d lekarski*.

[10] Cathcart S., Cantrell W., Elewski B. (2009). Onychomycosis and diabetes. *Journal of the European Academy of Dermatology and Venereology*.

[11] Sun C., Sun L., Ma H. (2012). The phenotype and functional alterations of macrophages in mice with hyperglycemia for long term. *The Journal of Cellular Physiology*.

[12] Limb G. A., Chignell A. H., Green W., LeRoy F., Dumonde D. C. (1996). Distribution of TNF*α* and its reactive vascular adhesion molecules in fibrovascular membranes of proliferative diabetic retinopathy. *British Journal of Ophthalmology*.

[13] Kolluru G. K., Bir S. C., Kevil C. G. (2012). Endothelial dysfunction and diabetes: effects on angiogenesis, vascular remodeling, and wound healing. *International Journal of Vascular Medicine*.

[14] Campos M. R. M., Russo M., Gomes E., Almeida S. R. (2006). Stimulation, inhibition and death of macrophages infected with *Trichophyton rubrum*. *Microbes and Infection*.

[15] Tacke F., Randolph G. J. (2006). Migratory fate and differentiation of blood monocyte subsets. *Immunobiology*.

[16] Geissmann F., Auffray C., Palframan R. (2008). Blood monocytes: distinct subsets, how they relate to dendritic cells, and their possible roles in the regulation of T-cell responses. *Immunology and Cell Biology*.

[17] Passlick B., Flieger D., Loms Ziegler-Heitbrock H. W. (1989). Identification and characterization of a novel monocyte subpopulation in human peripheral blood. *Blood*.

[18] Geissmann F., Jung S., Littman D. R. (2003). Blood monocytes consist of two principal subsets with distinct migratory properties. *Immunity*.

[19] Landsman L., Varol C., Jung S. (2007). Distinct differentiation potential of blood monocyte subsets in the lung. *The Journal of Immunology*.

[20] Nahrendorf M., Swirski F. K., Aikawa E. (2007). The healing myocardium sequentially mobilizes two monocyte subsets with divergent and complementary functions. *The Journal of Experimental Medicine*.

[21] Myśliwska J., Smardzewski M., Marek-Trzonkowska N., Myśliwiec M., Raczyńska K. (2012). Expansion of CD14^+^CD16^+^ monocytes producing TNF-*α* in complication-free diabetes type 1 juvenile onset patients. *Cytokine*.

[22] Venturini J., Golim M. A., Alvares A. M., Locachevic G. A., Arruda O. S., Arruda M. S. (2011). Morphofunctional evaluation of thymus in hyperglycemic-hypoinsulinemic mice during dermatophytic infection. *FEMS Immunology and Medical Microbiology*.

[23] Lenzen S. (2008). The mechanisms of alloxan- and streptozotocin-induced diabetes. *Diabetologia*.

[24] Rossini A. A., Like A. A., Chick W. L., Appel M. C., Cahill G. F. (1977). Studies of streptozotocin induced insulitis and diabetes. *Proceedings of the National Academy of Sciences of the United States of America*.

[25] Abd elaziz E. A. (2011). Pathological and biochemical studies on the effect of *Trigonella foenum*—Graecum and *Lupinus termis* in Alloxan induced diabetic rats. *World Applied Sciences Journal*.

[26] Venturini J., Álvares A. M., de Camargo M. R. (2012). Dermatophyte-host relationship of a murine model of experimental invasive dermatophytosis. *Microbes and Infection*.

[27] Russo M., Teixeira H. C., Marcondes M. C., Barbuto J. A. (1989). Superoxide-independent hydrogen peroxide release by activated macrophages. *Brazilian Journal of Medical and Biological Research*.

[28] Green L. C., Wagner D. A., Glogowski J., Skipper P. L., Wishnok J. S., Tannenbaum S. R. (1982). Analysis of nitrate, nitrite, and [15N]nitrate in biological fluids. *Analytical Biochemistry*.

[29] Breslin W. L., Strohacker K., Carpenter K. C., Haviland D. L., McFarlin B. K. (2013). Mouse blood monocytes: standardizing their identification and analysis using CD115. *Journal of Immunological Methods*.

[30] Moriguchi P., Sannomiya P., Lara P. F., Oliveira-Filho R. M., Greco K. V., Sudo-Hayashi L. S. (2005). Lymphatic system changes in diabetes mellitus: role of insulin and hyperglycemia. *Diabetes/Metabolism Research and Reviews*.

[31] Van Cutsem J., Janssen P. A. J. (1984). Experimental systemic dermatophytosis. *Journal of Investigative Dermatology*.

[32] Bosschaerts T., Guilliams M., Stijlemans B. (2010). Tip-DC development during parasitic infection is regulated by IL-10 and requires CCL2/CCR2, IFN-*γ* and MyD88 signaling. *PLoS Pathogens*.

[33] Geissmann F., Manz M. G., Jung S., Sieweke M. H., Merad M., Ley K. (2010). Development of monocytes, macrophages, and dendritic cells. *Science*.

[34] Hazra S., Jarajapu Y. P. R., Stepps V. (2013). Long-term type 1 diabetes influences haematopoietic stem cells by reducing vascular repair potential and increasing inflammatory monocyte generation in a murine model. *Diabetologia*.

[35] Sano H., Higashi T., Matsumoto K. (1998). Insulin enhances macrophage scavenger receptor-mediated endocytic uptake of advanced glycation end products. *The Journal of Biological Chemistry*.

[36] Giacco F., Brownlee M. (2010). Oxidative stress and diabetic complications. *Circulation Research*.

[37] Scivittaro V., Ganz M. B., Weiss M. F. (2000). AGEs induce oxidative stress and activate protein kinase C-*β*(II) in neonatal mesangial cells. *The American Journal of Physiology—Renal Physiology*.

[38] Moriello K. A., Hondzo H. (2014). Efficacy of disinfectants containing accelerated hydrogen peroxide against conidial arthrospores and isolated infective spores of *Microsporum canis* and *Trichophyton* sp.. *Veterinary Dermatology*.

[39] MacCarthy K. G., Dahl M. V. (1989). Inhibition of growth of *Trichophyton rubrum* by the myeloperoxidase-hydrogen peroxide-chloride system. *Journal of Investigative Dermatology*.

[40] Liu H.-F., Zhang H.-J., Hu Q.-X. (2012). Altered polarization, morphology, and impaired innate immunity germane to resident peritoneal macrophages in mice with long-term type 2 diabetes. *Journal of Biomedicine and Biotechnology*.

